# Bronchobiliary fistula

**DOI:** 10.4103/0972-9941.45208

**Published:** 2008

**Authors:** Ajay Mandal, Sanjay Sen, Sarfaraz Jalil Baig

**Affiliations:** Department of Surgery, The Calcutta Medical Research Institute. Calcutta-700 027, India

**Keywords:** Biliptysis, bronchobiliary fistula

## Abstract

Bronchobiliary fistula is a very rare complication of liver abscess. It presents with biliptysis (bile in cough), and chronic cough. Here we present a case of intractable biliptysis from a bronchobiliary fistula secondary to a liver abscess with biliary obstruction.

## INTRODUCTION

Bronchobiliary fistulas are very rare. In most cases, they are caused by hepatic or subphrenic abscesses, resulting from different conditions. It is a rare cause of chronic cough[1] leading to biliptysis. It is usually diagnosed by clinical history and imaging (CT/MRI). Treatment is usually endoscopic or surgery.

## CASE REPORT

A 55-year-old gentleman presented with the chief complaints of right upper abdominal pain along with fever with chills and rigor, and associated nausea and vomiting. On examination, there was mild icterus and tender right upper abdomen with guarding. Ultrasonography (USG) showed multiple hypoechoic space occupying lesions (SOL) in both lobes of the liver, largest one being 8 cm in diameter at the junction of both the lobes adjacent to the diaphragm. USG also showed dilated common bile duct CBD (11 mm) with a large stone obstructing the lower end. Hematological studies showed elevated bilirubin, liver enzymes, and leukocyte count. The provisional diagnosis was multiple pyaemic liver abscesses due to choledocholithiasis with cholangitis.

The patient was put on intravenous antibiotics. USG guided percutaneous transhepatic drainage of the largest SOL was done and an indwelling pig-tail catheter was placed in the cavity. An endoscopic retrograde cholangiography ERC with endoscopic sphincterotomy was done and the CBD was cleared of the stone. There was rapid recovery following this. The catheter initially drained pus and later bile which gradually decreased from 125 ml/day to 40 ml/day. Patient was sent home in a satisfactory condition with the indwelling catheter in situ and advice to report after a week. At home, after a few days, the catheter came off accidentally which he did not report or took seriously. A week later the patient started suffering from intractable cough with copious expectoration of green colored sputum which increased during sleep. The patient mistook the symptoms for respiratory infection as did the physicians he attended in his locality. Patient returned after a month to us with this presentation and was readmitted and investigations were carried out.

The sputum tested positive for bile. The USG showed a dilated CBD (14 mm) with sludge and heterogeneous echo pattern of the liver near the dome. Chest X-ray showed pleural effusion. Further imaging could not be done due to unaffordability.

The provisional diagnosis was biliptysis due to brochobiliary fistula secondary to residual bile duct obstruction.

**Figure 1:**
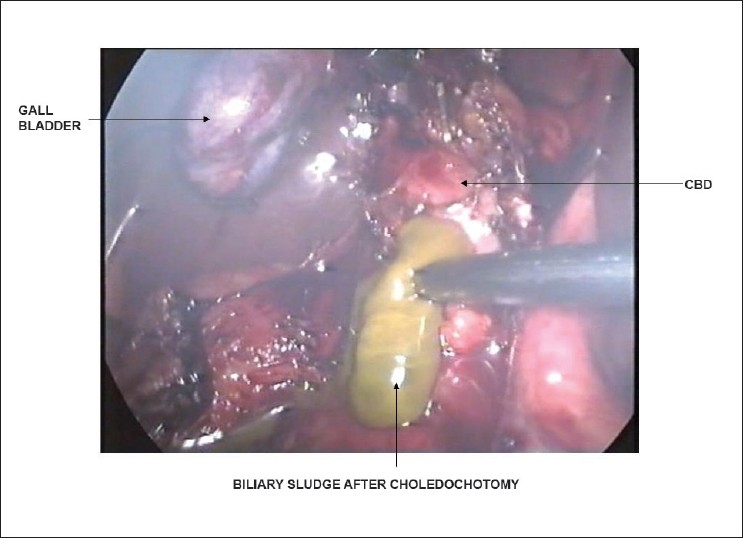
CBD was full of inspissated biliary sludge and tiny, crumbly, blackish calculi

**Figure 2:**
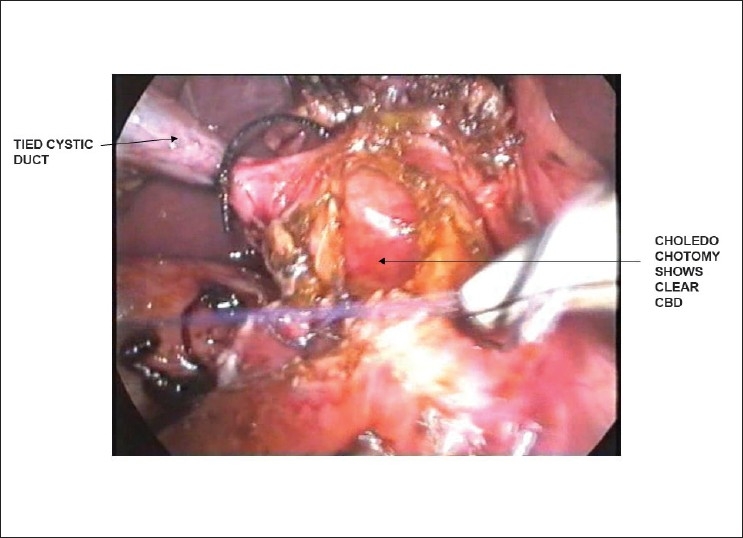
Choledochodueodenostomy in progress

Patient was subjected to laparoscopic exploration. Dome of the right lobe of liver was densely adherent to diaphragm and was not disturbed. The CBD was full of inspissated biliary sludge [Figure 1] and tiny, crumbly, blackish calculi. CBD was cleared and a choledochoduodenostomy [Figure 2] was done. The choledochoduudenostomy was done as a single layer interrupted sutures with 3-0 polyglactin. Concomitant cholecystectomy was added. The postoperative recovery was uneventful. The coughing gradually subsided over the next few days. The patient is doing well on 7 months follow up.

## DISCUSSION

A bronchobiliary fistula (BBF) is an uncommon entity with biliptysis being a pathognomonic sign. BBF is difficult to diagnose and requires a high clinical index of suspicion.

A BBF may be caused by liver abscess,[2] hepatic hydatids,[3] hepatic tumors, following radiofrequency thermal ablation of hepatic tumors, post liver resection, chronic pancreatitis and rarely as a late complication of transcatheter arterial embolisation (TAE).

In most cases, they are caused by hepatic or subphrenic abscesses, resulting from different conditions. Liver abscess with or without biliary lithiasis,[4] as the cause of BBF has been reported in literature. In our case, the cause was due to liver abscess secondary to stricture caused by biliary lithiasis.

They usually present with chronic cough, biliptysis, fever, and pain. Our case presented with intractable cough and biliptysis

Magnetic resonance cholangiography[5] may reveal a communication between the biliary tree and the bronchial tree. Hepatobiliary scintigraphy,[6] which is routinely used to visualize the liver and biliary tree, has been used as a noninvasive means for the precise diagnosis of a BBF.

BBF can be treated endoscopically or surgically. Endoscopic biliary sphincterotomy and repeated insertion of large size biliary plastic stents[7] have been used successfully for treatment of the fistula. Biliary lithiasis is extremely amenable to endoscopic management. We, in our case, did not do endoscopic treatment because patient opted for a single laparoscopic procedure of biliary obstruction. Endoscopic treatment in the form of nasobiliary drainage is a viable alternative to surgical treatment of BBF. Surgery in the form of resection of the involved pulmonary tissue and interposition of viable tissue between the lung and the fistulous tract is invasive but rapid resolution of the patients’ problem.[8]
